# Hydrophobically Modified Glucan as an Amphiphilic Carbohydrate Polymer for Micellar Delivery of Myricetin

**DOI:** 10.3390/molecules24203747

**Published:** 2019-10-17

**Authors:** Weiyu Yang, Ling Guo, Fenfen Li, Xin Liu, Shaoping Nie, Mingyong Xie, Danfei Huang

**Affiliations:** State Key Laboratory of Food Science and Technology, China-Canada Joint Lab of Food Science and Technology (Nanchang), Nanchang University, Nanchang 330047, China; Yangweiyu667@163.com (W.Y.); guoling_ncu@163.com (L.G.); ncusklifenfen@163.com (F.L.); liuxin_daisy@126.com (X.L.); nie68@sina.com (S.N.); xmync@163.com (M.X.)

**Keywords:** phytochemicals, myricetin, amphiphilicity, micelle, self-assembly

## Abstract

Myricetin (Myr) is a phytochemical with many functional properties. However, its hydrophobicity, low bioavailability, and stability limit its application. In this study, octadecanoate oat β-glucan (OGE) was synthesized and gained recognition as a self-assembled micelle forming a polymer with a critical micelle concentration (CMC) of 59.4 μg/mL. The Myr-loaded OGE micelle was then prepared and characterized by dynamic light scattering (DLS), transmission electron microscope (TEM), X-ray diffractometer (XRD), and Fourier-transform infrared spectroscopy (FT-IR) spectra. The water solubility of Myr was greatly enhanced by forming the Myr/OGE inclusion complex. Consequently, compared to free Myr, the retention of Myr in Myr-loaded OGE micelle was effectively increased during the intestinal digestion phase, and its antioxidant activity was also improved. Overall, our findings demonstrated the potential applications of OGE polymer for the development of prospective micelle in health food, cosmetics, and pharmaceutical fields because they can aid in the delivery of hydrophobic functional compounds like Myr.

## 1. Introduction

Myricetin (Myr) is a natural bioflavonoid that widely exists in plants, including tea, berries, fruits, vegetables, and medicinal herbs [1]. It possesses a wide range of biological activities, such as antioxidant [2], anticancer [3], antidiabetic [4], and anti-inflammatory activities [5]. However, its poor water solubility and susceptibility to degradation at high temperatures or certain pH are the most major challenges in utilizing its full potential [6]. To overcome these issues, different delivery carriers were used to achieve controllable Myr release. For example, Wang and his co-workers achieved the delivery of Myr across the blood-brain barrier using pluronic-modified mixed micelle [7]. Yao and his co-workers used hydroxypropyl-β-cyclodextrin and chitosan/β-glycerol phosphate nanogels/gels as intermediates to deliver Myr [8,9]. However, significantly shortened mean residence time (MRT) in plasma (*p* < 0.05) and a burst release of Myr from in vitro dissolution (over 98% within 10 min) were also observed and would probably cause potential side effects [8]. Thus, there is a need to develop better-tolerated and less toxic carriers for Myr delivery.

Over the last decades, amphiphilic polymers have attracted wide attention due to their advantages of enhancing water solubility and reducing side effects of hydrophobic components [10]. Among different materials being explored for the amphiphilic polymers, nature polysaccharides are highly stable, highly biocompatible, and lowly immunogenic. They also possess inherent bioactivities, such as facilitating mucoadhesion, enhancing targeting of specific tissues, and reducing the inflammatory response [11]. Furthermore, the hydrophilic nature of some polysaccharides can be exploited to enhance circulatory stability. After chemical modification, the hydrophobically modified polysaccharides can self-assemble because of the interaction of the hydrophobic groups and the hydrophilic chains [12,13]. Such systems are unique in having an extremely hydrophilic shell to adsorb hydrophilic molecules through non-covalent interactions and a hydrophobic core to encapsulate active ingredients with low water solubility. Natural polysaccharides have high hydrophilicity, non-toxicity, and good biocompatibility, which make them the best choice for amphiphilic carriers [14]. The modification of polysaccharides is relatively simple because of the existence of many functional groups in the structure of polysaccharides, such as hydroxyl, carboxyl, and amino groups [15,16]. Many hydrophilic polysaccharides (such as cellulose, inulin, mannose, and hyaluronic acid) have been reported for the preparation of micelle-like aqueous self-assemblies by hydrophobic modification with fatty acid chlorides, fatty acid methyl esters, aliphatic anhydrides, alkyl epoxides, or alkyl isocyanates, et al. [17,18,19]. 

Soluble β-glucan, derived from oats, is a linear polymer of glucose subunits connected by intrachain glycosidic *β-(1→3)* and *β-(1→4)* linkages. It was reported to improve the immune system [20], exhibits anticancer activity [21], and reduces blood cholesterol [22,23], lipids, and blood glucose [24,25]. To our best knowledge, the hydrophobic modification of β-glucan is still lacking. Therefore, esterification with stearic acid was adopted to prepare octadecanoate oat β-glucan (OGE). The present work focused on encapsulating myricetin within OGE micelles, and the complex properties were elucidated by dynamic light scattering (DLS), transmission electron microscopy (TEM), X-ray diffractometer (XRD), and Fourier-transform infrared spectroscopy (FT-IR) spectra. Then, the properties of Myr as a Myr/OGE inclusion complex, including solubility, retention rate, and antioxidate activities, were also evaluated.

## 2. Results and Discussion

### 2.1. OGE Synthesis

**Figure 1:**
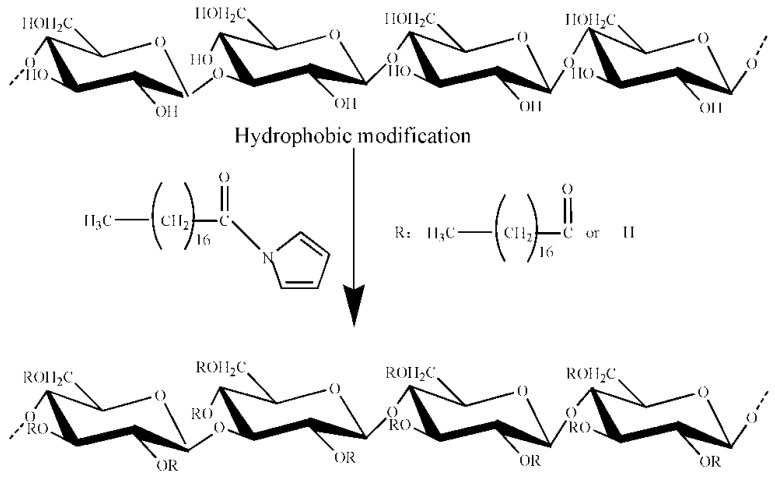
Preparation of octadecanoate oat β-glucan (OGE).

As shown in Figure 1, the amphiphilic OGE polymers were synthesized through the reaction between the carboxylic groups of stearic acid and the hydroxyl groups of β-glucan. The structure of the synthesized polymer was determined by FT-IR and ^1^H NMR. Data confirmed the presence of hydrophobic aliphatic modification of native β-glucan in OGE while keeping the poly-glucose backbone (The date was presented in Supplementary Materials).

For non-ionic dextran, the presence of hydrophilic groups is easy to graft onto the main chain of hydrophobic groups [15]. CDIs are widely used in most special esterification. During the preparation of the fatty acid activation solution, the hydrogen bond of stearic acid was broken by the catalytic action of CDI, and the imidazyl were then grafted onto the main chain of stearic acid to continue the reaction with oat β-glucan as an intermediate product. The imidazole group grafted onto the stearic acid was detached, and the corresponding stearic acid was also bound to the hydrogen bond of oat β-glucan in our study. In this way, the oat β-glucan was successfully grafted onto the hydrophobic stearic acid main chain to form amphiphilic polysaccharides in the reaction system. 

### 2.2. CMC Determination

**Figure 2:**
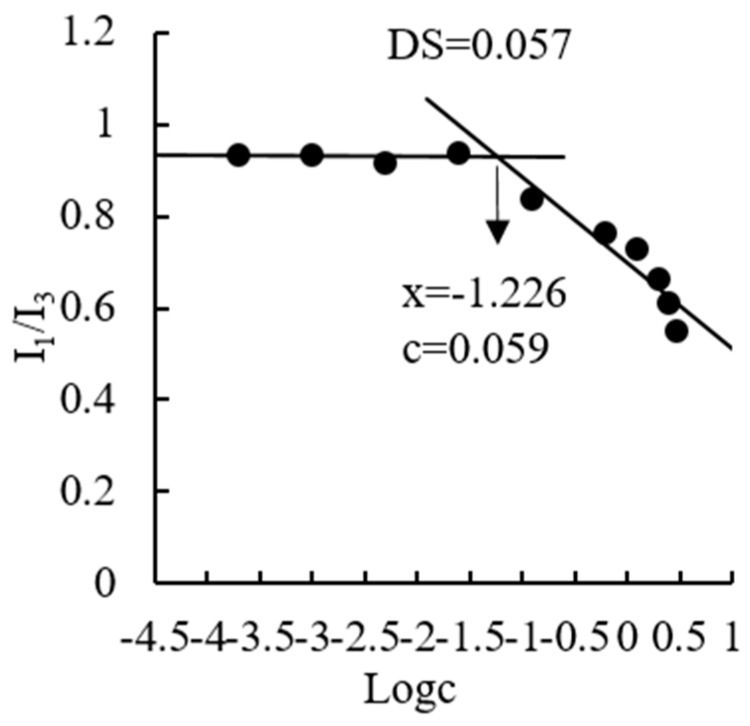
Plots of pyrene I_1_/I_3_ ratio versus the logarithm of different concentrations of OGE (DS = 0.057).

The CMC is the threshold concentration of self-aggregation and a crucial factor that determines the stability and accessibility of the formulation of polymeric micelles. In this study, the CMC of OGE was determined using pyrene as a fluorescent probe. Pyrene is poorly soluble in a polar environment (water) but solubilizes into the hydrophobic core of the micelles. When coexisting with polymeric micelles, the intensity of total emission, especially the third highest vibrational band at 385 nm (I_3_), of pyrene starts to drastically increase at a certain concentration of polymeric micelles. Thus, a high and low I_1_/I_3_ ratio indicates the polar and nonpolar microenvironments, respectively. A decrease in I_1_/I_3_ ratio will indicate that pyrene is transferred from an aqueous media (polar environment) to less polar microdomains induced by OGE micellization. Therefore, CMC is the concentration at the intersection of two straight lines between one fitted at low polymer concentration and the other fitted on the rapid rising of the graph. For OGE copolymer, I_1_/I_3_ values remained almost unchanged at lower concentrations (Figure 2). As concentration increases, the intensity ratio started to decrease, implying the onset of micelle aggregation. The CMC value of OGE, with degrees of substitution (DS) of 0.057, was calculated to be 59.4 μg/mL. This value was smaller than that of other low molecular weight surfactants (e.g., 0.061–0.081 mg/mL [26]).

### 2.3. Preparation and Characterization of Myr-Loaded OGE Micelle Complex 

#### 2.3.1. Preparation 

Amphiphilic polysaccharides, which could form self-aggregating micelles, have a shell-core structure [13]. When the myricetin is dispersed in the aqueous solution of amphiphilic polysaccharides, the myricetin was absorbed accordingly in the hydrophobic structure of the inner shell. Amphiphilic micelles are in a state of motion by continuous stirring in the process, and the non-covalent bonding can make myricetin enter the hydrophobic region better and form a wrapping state in the system. Meanwhile, the myricetin exposed in the aqueous solution is also protected.

#### 2.3.2. FT-IR Analysis

**Figure 3:**
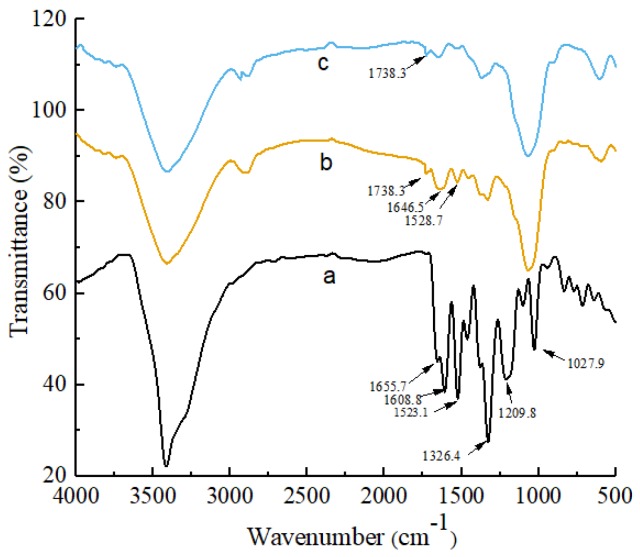
The FT-IR spectra of raw Myr (**a**), Myr-loaded OGE micelle complex (**b**), and OGE micelles (**c**).

FT-IR spectroscopy is a reliable technique for elucidating the structure, physical properties, and interactions of natural polymers. The FT-IR spectra of the Myr, OGE, and Myr-loaded OGE complex are shown in Figure 3. For Myr, the band at 3415.7 cm^−1^ corresponded to the stretching vibration of O-H, whereas the peaks at 1655.7 cm^−1^ and 1608.8 cm^−1^ were assigned to the stretching vibration of the C=O group that appears as a very strong doublet. The peak 1523.1 cm^−1^ was assigned to an aromatic group, and the peaks at 1326.4 cm^−1^ and 1209.8 cm^−1^ were attributed to the stretching vibration of C-O-C group [27]. The spectrum of OGE can be characterized by the band at 1738.3 cm^−1^, corresponding to the vibration of the ester carbonyl groups, and some other prominent peaks at 3415 cm^−1^(-OH), 2929 cm^−1^(C-H), and 890 cm^−1^ (β-configuration) [28]. However, in the spectra of the Myr-loaded OGE complex, some characteristic bands of Myr were shifted or absent. Compared with Myr spectrum, the disappearance of the absorption bands of C=O (at 1655.7 cm^−1^ and 1608.8 cm^−1^) and C-O-C (at 1326.4 cm−1 and 1209.8 cm^−1^) vibration indicated that the vibration of these three groups on C rings might have been restricted due to the formation of the inclusion complex. In addition, signals from aromatic groups (1523.1 cm^−1^) were slightly shifted to 1528.7 cm^−1^ and greatly weakened, indicating that a majority of hexatomic rings of Myr were included within OGE micelles. The result of FT-IR confirmed the successful inclusion of Myr into OGE micelles.

#### 2.3.3. PXRD Analysis

**Figure 4:**
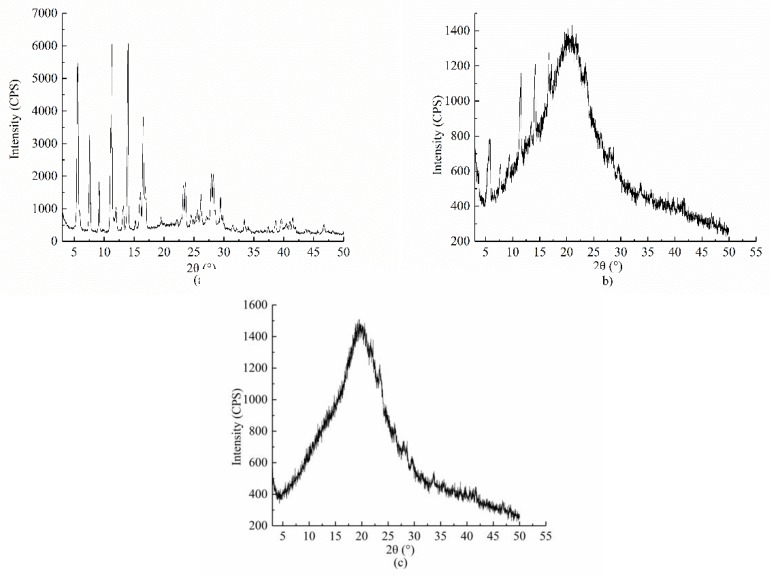
Powder X-ray diffractogram for (**a**) Myr, (**b**) physical mixing of Myr and OGE, and (**c**) Myr loaded OGE micelles.

The crystalline states of Myr, physical mixing of Myr and OGE, and Myr loaded OGE micelles were examined by powder X-ray diffraction and are illustrated in Figure 4. Free Myr exhibited three strong characteristic diffraction peaks at 5.8°, 13.2°, and 14.9°. After physical mixing with OGE, these characteristic peaks of Myr remained apparent. However, as to the Myr loaded OGE micelles, there only remained an amorphous peak around 23°, which was ascribed to the characteristic peaks for the poly-glucose chain. The amorphous peak was at the same degree as the blank OGE micelles, indicating that the Myr loaded OGE micelle complex was successfully prepared, and the crystal structure of Myr entered the core of OGE and formed a wrapped structure.

#### 2.3.4. Size and PDI Measurements

**Figure 5:**
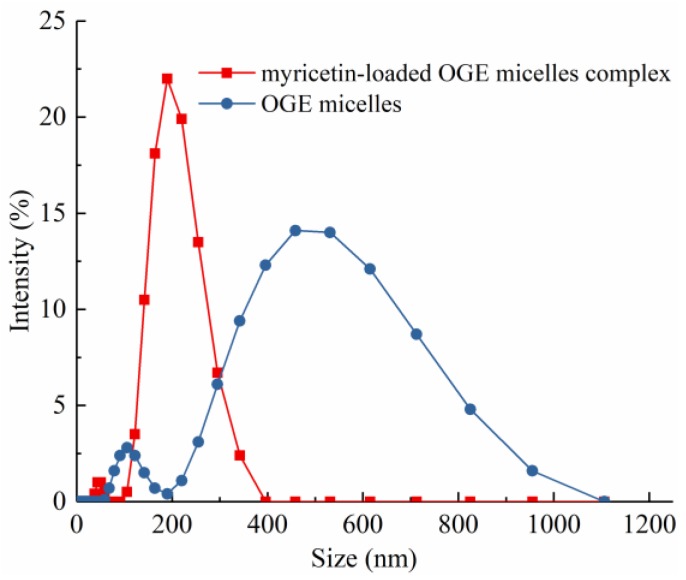
The intensity of size distribution of Myr-loaded OGE micelle complex and OGE.

The size of polymeric micelles and their size distribution in aqueous media were measured by dynamic light scattering (DLS). Since the unmodified β-glucan does not form micelles, it cannot be used as a control. The polydispersity index (PDI) of OGE micelles and Myr-loaded OGE micelles were 0.48 ± 0.03 and 0.44 ± 0.05, respectively. The mean diameter of OGE micelle in water was 486.43 ± 23.73 nm and significantly decreased after the encapsulation of Myr into the micelles (Table 1). This could be attributed to the increase of hydrophobicity of the system, which lead to the formation of a tighter hydrophobic core interaction between Myr and OGE after Myr was loaded in. Our observation was similar to various reported studies involving hydrophobically modified carbohydrates as micelle forming polymers. It is interesting to observe that a narrow, monomodal particle size distribution was obtained when Myr was loaded in, which indicated that the nanoparticles in this system became homogenous (Figure 5).

#### 2.3.5. Transmission Electron Microscope (TEM)

**Figure 6:**
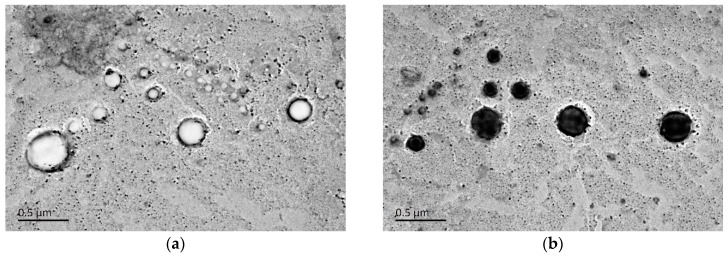
TEM image of (**a**) OGE and (**b**) Myr-loaded OGE micelles.

From the TEM image, as shown in Figure 6, it was observed that both OGE and Myr-loaded OGE micelles had a spherical or ellipsoidal morphology. The middle core of OGE micelles displays as hollow while the stained Myr-loaded OGE micelles appear as dark objects against the light background of the amorphous carbon substrate, which indicated the incorporation of Myr into micelles. The particle size of OGE and Myr-loaded OGE micelles measured by TEM (around 200 nm) was smaller than the results confirmed by DLS. This may be due to the different preparation methods of the sample, the difference in the interaction between the molecules, and the state of the sample at the time of measurement or observation. Besides, some particles with a diameter of around 100–150 nm were also observed under TEM, which may be caused by different DS of octadecanoate to oat β-glucan.

### 2.4. Dissolubility Study 

**Figure 7:**
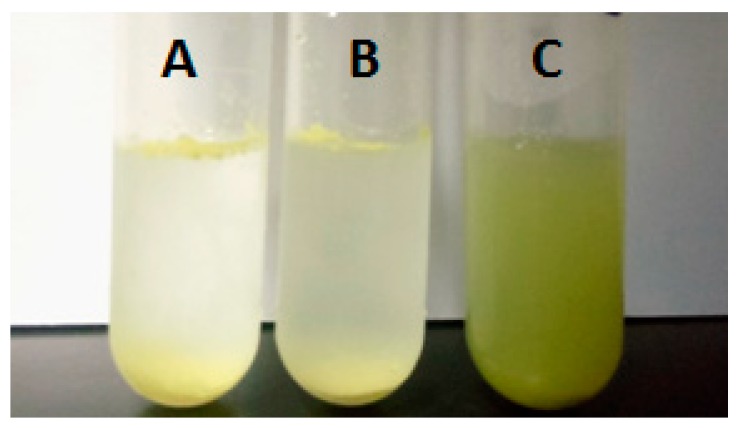
Photographic image of Myr dissolved in different solutions (**A**: water; **B**: oat β-glucan solution; **C**: OGE micelle solution).

The dissolubility profiles of samples (Myr, Myr-loaded OGE micelle complex, and the physical mixture of Myr and β-glucan, that contain an equivalent dose of Myr) are shown in Figure 7. The loaded Myr concentration in aqueous 1.5 mg/mL OGE solution was measured at 83.79 μg/mL, which was 8-fold of its solubility in water (9.93 μg/mL). The solution of the Myr-loaded OGE micelle complex showed a clear, well-dispersed formulation with the natural color of Myr. However, an equal weight of free Myr appeared as insoluble particulates with visible precipitates and some suspension. As shown in Table 2, the solubilization of Myr in the presence of β-glucan was 17.63 μg/mL, which was much lower than that of OGE micelles (83.79 μg/mL) and consistent with the photographic image. Therefore, our results suggested that OGE self-assemblies are very efficient in solubilizing Myr.

### 2.5. In Vitro Retention Rate Studies

**Figure 8:**
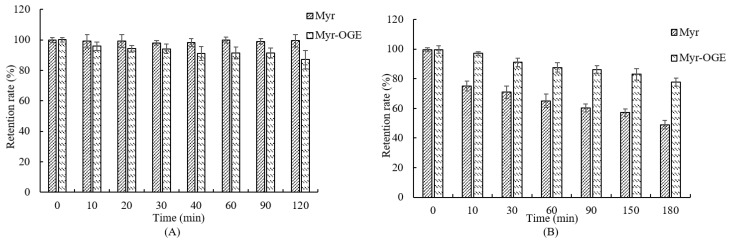
The retention rate of Myr in simulated gastric (**A**) and intestinal digestion (**B**). Each value is the means ± standard deviation (SD) of triplicate measurements.

As well acknowledged, the degradation of Myr, which is a weak acidic compound, is highly sensitive to the mild alkaline conditions [29]. A good proportion of the Myr can be transformed into other unknown and/or undetected components with different chemical properties and, consequently, different bioavailability and biological activity. Hence, the in vitro retention characteristics of Myr in OGE micelles were investigated in simulated gastric (pH 2.0) and intestinal (pH 7.0) digestive fluid, respectively. Free Myr retained in DMSO was investigated as a control. As shown in Figure 8A, a high retention rate of free Myr in the simulated gastric digestive fluid was observed, which might be due to its high stability at a low pH value. However, the retention rate of Myr in OGE micelles slowly decreased with the extension of time in the simulated gastric digestive fluid. This is probably due to the interaction among Myr, OGE, and digestive enzymes. As to simulated intestinal digestive fluid (pH 7.0) (Figure 8B), the retention rate of free Myr decreased rapidly in the first 10 min and more than half in 3 h. In contrast to free Myr, the retention rate of Myr in OGE micelles could still reach 75.98% at 3 h, and the slower decline trend of the retention rate indicates that the OGE micelles are capable of providing a sustained release of Myr.

### 2.6. Antioxidant Activities

**Figure 9:**
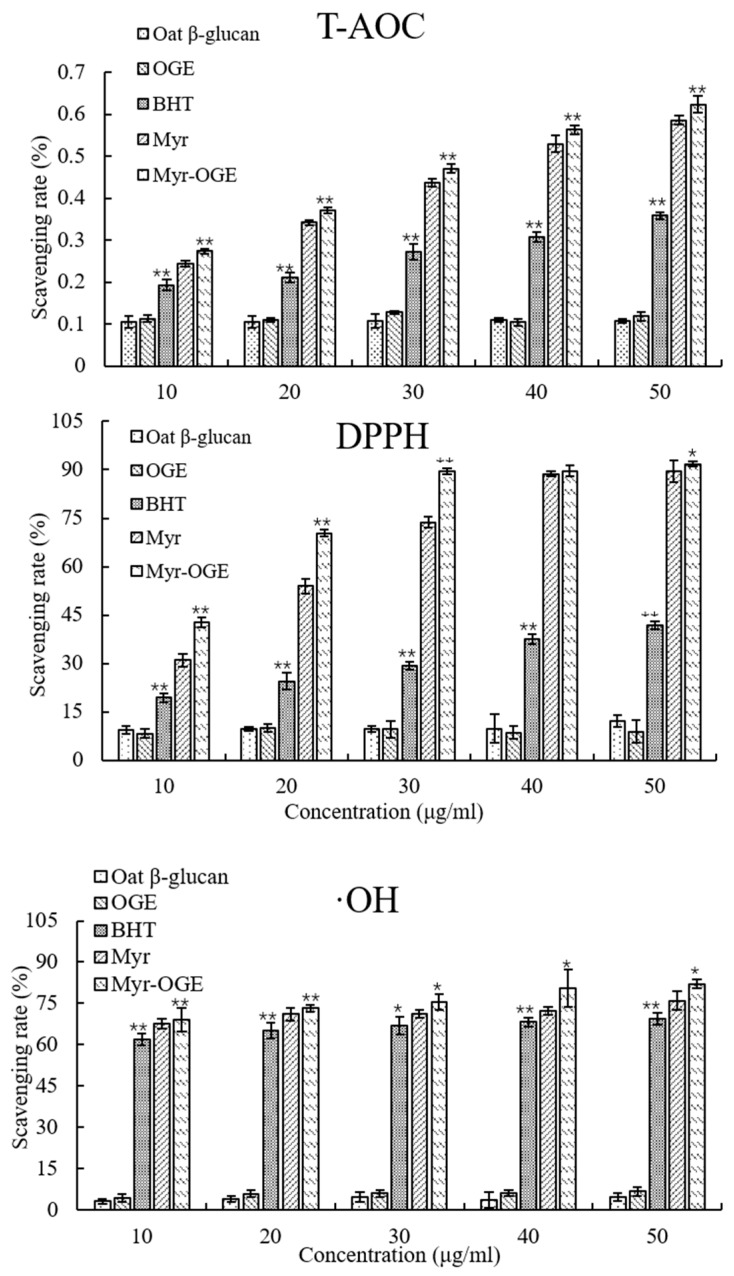
Antioxidant activities of Oat β-glucan, OGE, free Myr, Myr loaded OGE micelles in vitro. (**A**) Total antioxidant capability, (**B**) DPPH radical scavenging activity, and (**C**) hydroxyl radical scavenging activity. Each value is the means ± standard deviation (SD) of triplicate measurements. ** *p* < 0.01 compared with free Myr; * *p* < 0.05 compared with free Myr.

The antioxidant activities of Myr in the micelles were quantified via radical-scavenging activity measurement and ORAC assay and were then compared to those of the free Myr solution. As shown in Figure 9, Oat β-glucan and OGE did not show any antioxidant activities in the selected concentration gradient. Compared to free Myr, the Myr loaded OGE micelle complex has significant enhanced DPPH· radical scavenging activity in the selected concentration gradient, particularly at concentrations lower than 30 μg/mL. Both the radicals scavenging activity of free Myr and its complex were concentration dependent within the evaluated range. Regarding the hydroxyl radicals scavenging activity, free Myr solution and encapsulated Myr in micelles exhibited stronger radical scavenging activity than did the positive control BHT. While the radical scavenging activity in the free Myr solution was significantly lower than that of encapsulated Myr in micelles. Regarding the total antioxidant capacity, the result was similar to that of the DPPH·radicals scavenging activity: the total antioxidant capacity increased with the increasing concentration in BHT, free Myr solution and encapsulated Myr in micelles. Compared with the free Myr solution, an improved antioxidant activity could be observed in encapsulated Myr in micelles. According to reports, raw Myr has poor aqueous solubility that may lead to the incomplete dispersion in the solvents. Myr molecules could not fully interact and react with the oxidants or free radicals. Thus, we can speculate that the increases in the antioxidant activity of the Myr-loaded OGE micelle complex can likely be attributed to its enhanced solubility.

## 3. Materials and Methods 

### 3.1. Chemicals and Reagents

Commercial soluble β-glucan (80 wt% β-glucan content) extracted from oats was bought from Yikang Biological Technology Co., Ltd. (Zhangjiakou, China). Myr with a purity of greater than 98% (standard substance in HPLC analysis) was obtained from Wekeiqi Biotechnology Co. Ltd. (Sichuan, China). Stearic acid (analytical grade) was supplied by Sinopharm Chemical Reagent Co., Ltd. (Shanghai, China). Dimethyl sulfoxide (DMSO, analytical grade), Salicylic acid (analytical grade) were obtained from Damao Chemical Reagent Factory. N,N′-carbonyldiimidazole (99%), potassium bromide were purchased from Aladdin Reagent Co., Ltd. (Shanghai, China). Isopropanol (analytical grade), anhydrous ethanol (analytical grade), sodium chloride (analytical grade), potassium chloride (analytical grade), and sodium bicarbonate (analytical grade) were obtained from Xilong Scientific Co., Ltd. (Guangdong, China). 1,1-diphenyl-2-picrylhydrazyl (DPPH), Pepsin, and trypsin were provided by Sigma-Aldrich (St Louis, MO, USA). All other reagents used in the study were of analytical grade. 

### 3.2. Synthesis of OGE

OGE was prepared by modifying oat β-glucan with stearic acid, in brief, stearic acid was accurately weighted and dipped in 25 mL DMSO maintained at 70 °C in a water-bath until completely dissolved. Then, an equal amount of N,N′-carbonyldiimidazole was added and mixed evenly. After reaction at 70 °C for 15 h, acyl-imidazole stearate activating solution was obtained. A total of 1 g of β-glucan was dissolved in 20 mL DMSO, different amounts of acyl-imidazole stearate were added, and the mixture was incubated at different reaction temperatures for different times under continuous stirring. Reaction products were precipitated by adding isopropyl alcohol and then dissolved in distilled water. The residues were dried in a rotary evaporator (RE-2000A, Shanghai Yarong Biochemistry Instrument Factory, Shanghai, China) and the pellets were successively dialyzed against tap water, ultrapure water, and distilled water for 24 h in a dialysis bag (molecular weight cut-off: 8000–14,000 Da), respectively. A series of OGE with different degrees of substitution (DS) were obtained by drying with an ALPHA 1-2 freeze dryer (Martin Christ GmbH, Osterode im Harz, Germany) [30].

The values of DS was determined referring to previous reports [30,31] and slightly modified. A certain amount of OGE was completely dissolved in 0.50 mL DMSO, adding 1 mL 4 mg/mL prepared sodium methanol solution, and heating it airtight at 80 °C for 1 h. After the sample cooled, 1 mL n-heptane and 1 mL saturated sodium chloride solution was added, and the supernatant was filtered (0.22 μm). The content of methyl stearate was determined by GC (6890N, with FID Detector and Rev.A.10.02 Chromatography Workstation Agilent Technologies, Inc., Santa Clara CA, USA). The determination conditions were as follows: CP-Sil88 capillary column (100 m × 0.25 mm × 0.39 mm, 0.20 um, Varian Inc., North Charleston, SC, USA), FID detector temperature 250 °C, sample inlet temperature 250 °C.

DS = 162 × A%/((M × 100 − (M − 1) × A%)(1)

Here, 162 is the molecular weight of the anhydroglucose unit, A is the content of the acyl group, and M is the molecular weight of the acyl group.

### 3.3. Preparation of OGE Micelles 

OGE (7.5 mg) with a DS of 0.057 was dissolved in 5 mL of distilled water by heating at 100 °C for 2 min. The solution was cooled to room temperature when OGE was completely dissolved, and then equilibrated by stirring at 100 r/min for 24 h at room temperature [32]. The obtained OGE micelles were used for characterization or loading Myr.

### 3.4. Determination of Critical Micelle Concentration (CMC)

The critical micelle concentration (CMC) of amphiphilic OGE polymer in aqueous media was measured using pyrene as a fluorescence probe. Briefly, 20 μL pyrene/methanol solutions (3 × 10^−4^ M) were added into a tube and blow-dried with nitrogen to remove methanol. Following this, 10 mL of OGE micelles solutions with various concentrations (0.0002–3 mg/mL) was added, and the solution was incubated at room temperature under high speed stirring for 5 h. The final concentration of pyrene was 6.0 × 10^−7^ M. Fluorescence spectra were recorded on a fluorescence spectrometer (F-700, Hitachi, Japan). The emission spectra were recorded, ranging from 350 nm to 500 nm, with an excitation wavelength at 330 nm. The fluorescence intensity ratios of the first peak at 374 nm to the third peak at 385 nm (I_1_/I_3_) were calculated and plotted against the logarithm concentration of OGE micelles [33]. The intersection point of the two straight lines was determined, and the CMC was calculated.

### 3.5. Preparation of Myr Loaded OGE Micelles

The inclusion complex of Myr and OGE was prepared by the suspension method [34]. A total of 7.5 mg of Myr was added into 5 mL OGE micelles solution containing 7.5 mg of OGE (the ratio of Myr to OGE weight was 1:1). Then, the solution was homogenized at 12,000 rpm for 3 min using a homogenizer (FJ200-SH, Shanghai Specimen and Model Factory, Shanghai, China). The mixture was then continuously stirred at room temperature for 41 h to make a clear solution. After standing for some time to remove the undissolved raw material, a Myr-loaded OGE micelles solution was obtained.

### 3.6. Characterization of Myr-Loaded OGE Micelles

#### 3.6.1. Fourier-Transform Infrared Spectroscopy (FT-IR)

FT-IR spectra were recorded using a Nicolet FT-IR 5700 spectrophotometer (Thermo Fisher Scientific, Waltham, MA, USA). The samples of raw myricetin or myricetin-loaded OGE micelles were previously ground and mixed thoroughly with KBr at a ratio of 1:100. The KBr disks were prepared by compressing the powder, and the wavelength region was between 4000 cm^−1^ and 400 cm^−1^.

#### 3.6.2. Crystallinity Analysis Under X-ray Diffractometer (XRD)

The XRD pattern of the myricetin reference substance and MYR-OGE was measured by an X-ray diffractometer (D8 ADVANCE, BRUKER, Germany). For the measurement, the sample was irradiated at a voltage of 40.0 Kv and a current of 40.0 mA, wherein the measurement range is 3°–50°, and the scanning rate is 2°/min [8].

#### 3.6.3. Measurement of Size and PDI

The hydrodynamic diameter and polydispersity index (PDI) of the samples were measured via a dynamic light scattering (DLS) method, using a Malvern Zetasizer Nano (Malvern Instruments Ltd., Malvern, UK).

#### 3.6.4. Transmission Electron Microscope (TEM)

Morphological evaluation of myricetin, OGE, myricetin-loaded OGE micelle complex, and a physical mixture of myricetin and OGE was performed by TEM [35]. At least 5 pictures for each sample were taken at randomly selected locations.

### 3.7. Determination of Loading Capacity (LC)

Before conducting the analysis, the Myr-loaded OGE micelle solution was mixed with ethyl alcohol (1:3, *v*/*v*) to extract Myr. Mixtures were vortexed for 3 min and then ultrasonic treated for 3 min. After vigorous shaking at room temperature for 30 min and then centrifuging at 8000× *g*/min for 10 min, the resultant upper ethyl alcohol layer containing Myr was collected and determined using a UV-VIS Spectrophotometer (Varioskan Flash Multimode Reader, Thermo Fisher Scientific, USA) at 377 nm (obtained by full wavelength scanning, and the standard curve of myricetin is not listed). The Myr loading capacity (LC) was calculated using the following equation:

LC = Weight of Myr in micelles/Weight of OGE(2)

### 3.8. Dissolubility Study

The in vitro dissolution studies of Myr, Myr/β-glucan physical mixture, and Myr-loaded OGE micelle complex were conducted. In the present studies, samples equivalent to 2.5 mg of Myr were taken into glass tubes, then, 150 μL upper liquid were vortexed with ethyl alcohol and sonicated for 3 min, and then centrifuged at 8000× *g* for 10 min using a high-speed centrifuge. A 200 μL resultant upper ethyl alcohol layer was collected and determined at 377 nm. The solubility numerical values were calculated by introducing standard curves. 

### 3.9. Simulated Gastric and Intestinal Digestion In Vitro

#### 3.9.1. Simulated Gastric Digestion In Vitro

The gastric digestion was carried out according to the reported methods with some modifications [36]. Briefly, the simulated gastric fluid (SGF) consisted of 0.5 M KH_2_PO_4_, 0.5 M MgCl_2_·6H_2_O, 0.5 M (NH_4_)_2_CO_3_, 0.5 M KCl, 2 M NaCl, and 1 M NaHCO_3_. The final pH was adjusted to 2 by the addition of 6.0 M HCl. Then, 3.84 mg of pepsin from porcine gastric mucosa (3200–4500 U/mg) was added to a solution of 4.0 mL myricetin-loaded OGE micelle complex (2 mg·mL^−1^), 3.0 mL of SGF, and 2 μL of CaCl_2_ (0.3 M). The final pH was adjusted to 2 by the addition of 1.0 M HCl, and the final volume was brought to 8 mL with deionized distilled water. The gastric digestion of the samples and the control (water) were followed for 120 min. Samples were analyzed after 0, 10, 20, 30, 40, 60, 90, and 120 min of digestion. Three independently replicated extractions were performed for each sample. The stability of Myr-loaded OGE micelles in the stomach was expressed by the retention rate of Myr (RR%) using the following equation:

RR% = (Weight of Myr in micelles − Weight of Myr released into the simulated solution)/Weight of Myr in micelles × 100(3)

#### 3.9.2. Simulated Intestinal Digestion In Vitro

For the intestinal digestion test, 4 mL of the Myr-loaded OGE micelles complex solution predigested by gastric medium was mixed with 2.2 mL of simulated intestinal fluid (SIF) containing 0.5 M KH_2_PO_4_, 0.5 M MgCl_2_·6H_2_O, 0.5 M KCl, 2 M NaCl, and 1 M NaHCO_3_. To realistically simulate a duodenum residue solution, 8 μL of CaCl_2_ (0.3 M), 19.2 mg of bile salt, 8.0 mg of trypsin (the activity is equivalent to 4 × of USP specifications according to the US Pharmacopeia) was added. The final pH was adjusted to 7 by the addition of 1.0 M NaOH, and the final volume was brought to 8 mL with deionized distilled water. The gastric digestion of the samples and the control (water) were followed for 180 min. Samples were analyzed after 0, 10, 30, 60, 90, 150, and 180 min of digestion, and the stability of Myr-loaded OGE micelles complex in the intestinal tract was expressed by the retention rate of Myr using the equations listed above. Three independently replicated extractions were performed for each sample. 

### 3.10. Antioxidant Activities

Antioxidant activities of the Myr-loaded OGE micelles were estimated by total antioxidant capability (T-AOC), DPPH· and ·OH radicals scavenging assays.

#### 3.10.1. Scavenging Activity of DPPH·Radicals

OGE, β-glucan, and the Myr-loaded OGE complex were completely dissolved in pure water and free Myr (same to the Myr content in Myr-loaded OGE complex), and butylated hydroxytoluene (BHT, as a positive control) was dissolved in absolute ethanol. Then, 0.4 mL of a freshly prepared 0.2 mM DPPH solution was added to 0.1 mL of the samples with indicated concentration (10 μg/mL, 20 μg/mL, 30 μg/mL, 40 μg/mL, and 50 μg/mL) and 0.3 mL water. The mixture was shaken and kept for 30 min at 37 °C, and the absorbance was measured at 517 nm. All measurements were determined in triplicate, and the DPPH scavenging effect was calculated as follows:

Scavenging activity (%) = (1 − (A_1_ − A_2_)/A_0_)) × 100% (4)

Here, the solution of DPPH was used as a negative control A_0_, the mixture of DPPH solution and sample solution were used as A_1_, and the solution of samples were used as A_2_.

#### 3.10.2. Scavenging Activity of Hydroxyl Radicals

Scavenging hydroxyl radicals of polysaccharide samples were studied employing the modified method by Halliwell, Gutteridge, and Aruoma [37]. Briefly, OGE, β-glucan, Myr-loaded OGE complex, free Myr and butylated hydroxytoluene (BHT, as a positive control) were prepared as described above. 0.1 mL of sample solution was mixed with 0.1 mL of 2.5 mg/mL FeSO_4_, 0.1 mL of H_2_O_2_ (0.018%), and incubated at 37 °C for 10 min, 0.1 mL of 1.2 mg/mL salicylic acid were then added and the mixture was incubated at 37 °C for 30 min under dark conditions, and the absorbance were measured at 510 nm. All measurements were determined in triplicate, and the hydroxyl radicals scavenging effect was calculated as follows:

Scavenging activity (%) = (1 − (A_1_ − A_2_)/A_0_)) × 100% (5)

Here, the mixture of FeSO_4_, salicylic acid, and H_2_O_2_ solution was used as a negative control A_0_, the mixture of FeSO_4_, salicylic acid plus H_2_O_2_ solution, and sample solution was used as A_1_, and the mixture of FeSO_4_, salicylic acid, and sample solution was used as A_2_.

#### 3.10.3. Total Antioxidant Capability (T-AOC)

Total antioxidant capacity was determined using a commercial kit (Nanjing Jianchen Bioengineering Institute, Nanjing, China). The working fluid used in the experiment needs to be prepared at the time of use. After adding samples, the reaction system needed to be accurately reacted in the dark at room temperature for 6 min, and then the absorbance was read at 405 nm. The total antioxidant capacity of the sample was obtained by comparison with a standard curve.

### 3.11. Statistical Analysis

The results were expressed as mean ± standard deviation (*S.D.*) of the indicated number of experiments. The statistical significance was estimated using a Student’s *t*-test, *p* < 0.05 were considered as statistically significant, *p* < 0.01 as highly significant.

## 4. Conclusions

The hydrophobic modification of Oat β-glucan was synthesized by a simple esterification reaction, and then a Myr-loaded OGE micelle complex was successfully prepared. The physicochemical properties determined by FT-IR, XRD, DLS, and TEM showed the successful encapsulation of Myr into the cavity of OGE through non-covalent bonds. The aqueous solubility of the Myr-loaded OGE micelle complex was increased significantly compared to free Myr. Correspondingly, the retention and antioxidant activity of Myr in the Myr-loaded OGE micelle complex were greatly improved. In conclusion, the above results suggested that the self-aggregated micelle of OGE would be applied as promising hydrophobic nutrient delivery carriers in food and biomedical fields.

## Figures and Tables

**Table 1:** The size and PDI of Myr-loaded OGE micelles.

Sample	Size (nm)	PDI
**OGE micelles**	486.43 ± 23.73	0.48 ± 0.03
**Myr-loaded OGE micelle complex**	216.93 ± 41.40	0.44 ± 0.05

**Table 2:** The solubilization of Myr in different solutions under the same preparation conditions.

Sample	Solute	Solubility of Myr (μg/mL)
1	Aqueous solution	9.93 ± 2.51
2	Oat β-glucan aqueous solution	17.63 ± 5.91
3	OGE aqueous solution	83.79 ± 1.10

## Supplementary Materials

The following are available online, Figure S1: Octadecanoate oat β-glucan (OGE) by hydrogen nuclear magnetic resonance, Figure S2: The appearance of oat β-glucan and OGE under scanning electron microscope, Figure S3: Effect of the Degree of substitution (DS) of OGE in LC of Myr-OGE.
