# Supplementary material for: Hydrophobically Modified Glucan as an Amphiphilic Carbohydrate Polymer for Micellar Delivery of Myricetin
Source: Molecules. 2019 Oct 17;24(20):3747. doi: 10.3390/molecules24203747 (PMC6833000; doi:10.3390/molecules24203747)

Supplementary Materials

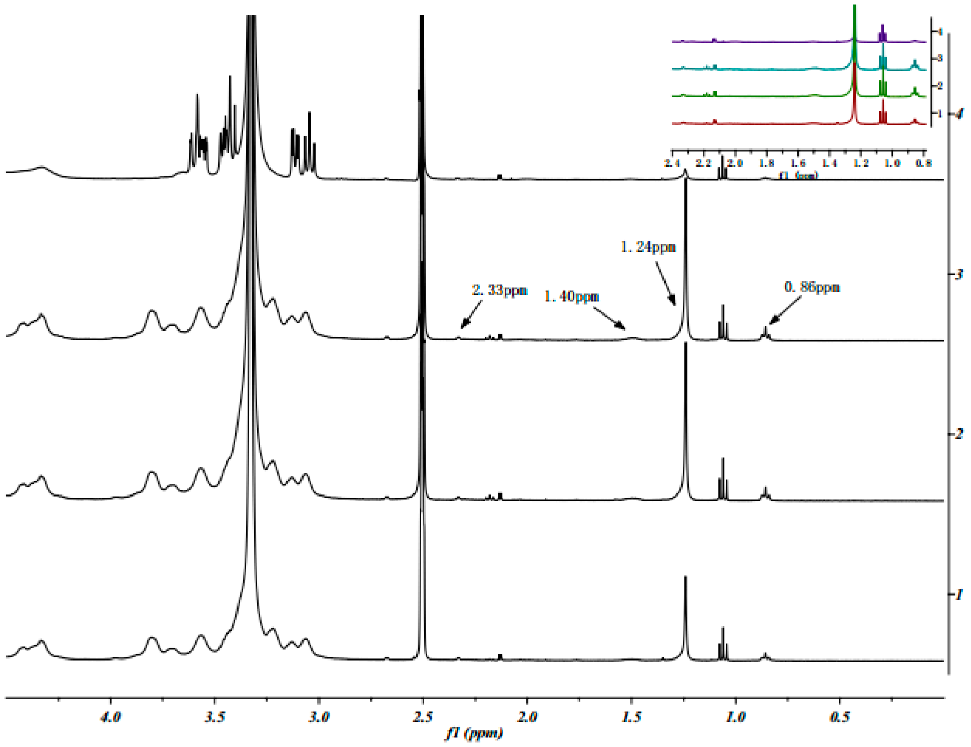

Figure 1. Octadecanoate oat β-glucan (OGE) by hydrogen nuclear magnetic resonance.

(1. OGE of DS = 0.021; 2. OGE of DS = 0.037; 3. OGE of DS = 0.040; 4. oat β-glucan)

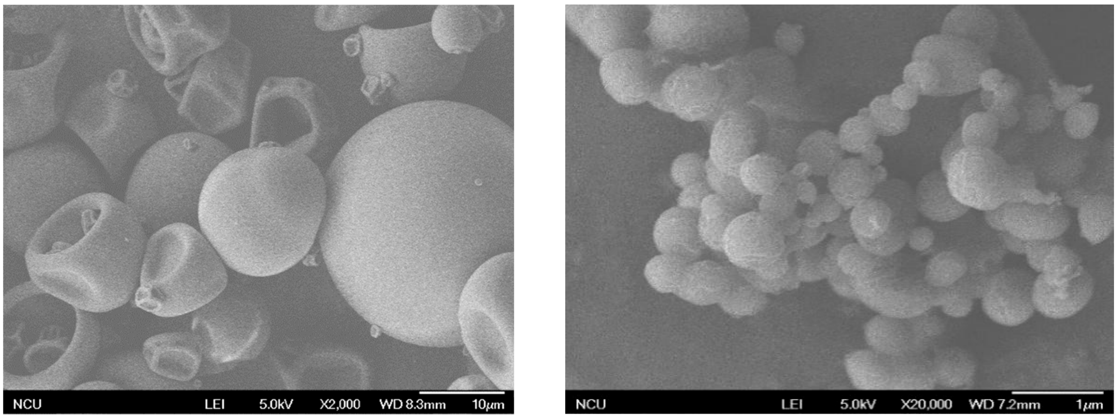

(A) (B)

Figure 2. The appearance of oat β-glucan and OGE under scanning electron microscope (**A**: oat β-glucan; **B**: OGE).

Effect of the Degree of substitution(DS) of OGE in LC of MYR-OGE

In this experiment, nine samples with different DS (DS = 0.021, 0.045, 0.057, 0.084, 0.088, 0.090, 0.102, 0.129, and 0.133) were selected. The ratio of MYR to OGE was 1:1, the homogenization rate was 12 kr/min, and 3 min of homogenization. The experiment was repeated three times and the measured values were recorded.

Determination of the optimal DS of the OGE

After selecting the nine different DS of OGE samples to load the myricetin, it was found that there was an inextricable relationship between the DS of OGE and the weight of the loaded myricetin. As seen in the figure, the weight of the loaded myricetin first increased with the increase of the DS of OGE. When the DS of OGE increased to 0.057, the effect of loading myricetin was the best. As the DS of OGE continued to increase, the weight of the loaded myricetin began to decrease. When the DS of OGE was 0.102, 0.129, and 0.133, there was no significant change in the loading effect of myricetin. It has also been shown that the highest substitution of OGE is not the best for the loading of myricetin. Therefore, OGE with DS = 0.057 was used as a carrier for this experimental research.

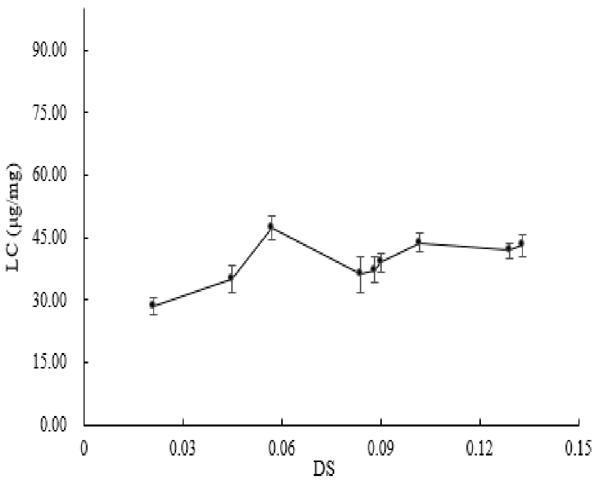

Supplement: Supplementary file 1 [file molecules-24-03747-s001.pdf]
